# Clinical Burden of Anastomotic Leak After Elective Colorectal Cancer Surgery: A Multicenter Cohort Study

**DOI:** 10.3390/jcm15176903

**Published:** 2026-09-06

**Authors:** Tomasz Cwaliński, Patryk Kaczor, Bartosz Kapturkiewicz, Maciej Ciesielski, Michał Kazanowski, Zuzanna Bigus, Jacek Piątkowski, Marcin Januszkiewicz, Paweł Lesiak, Sergii Girnyi, Damian Nowiński, Piotr Kurek, Marek Jackowski, Marek Bębenek, Mateusz Jagielski, Luigi Marano

**Affiliations:** 1 Department of General Surgery and Surgical Oncology, “Saint Wojciech” Hospital, “Nicolaus Copernicus” Health Center, 80462 Gdańsk, Poland; tcwalinski@copernicus.gda.pl (T.C.); sgirnyi@copernicus.gda.pl (S.G.); 2 Department of General, Gastroenterological and Oncological Surgery, Collegium Medicum, Nicolaus Copernicus University in Toruń, 87-100 Toruń, Poland; patryk.kaczor97@gmail.com (P.K.); jpiatkowski@wp.pl (J.P.); jackowscy@hotmail.com (M.J.); matjagiel@gmail.com (M.J.); 3 Faculty of Medicine, Wroclaw University of Science and Technology, 53-413 Wroclaw, Poland; bartosz.kapturkiewicz@dcopih.pl (B.K.); michal.kazanowski@dcopih.pl (M.K.); marek.bebenek@dcopih.pl (M.B.); 4 1st Department of Surgical Oncology, Lower Silesian Oncology, Pulmonology and Hematology Center, Pl. Hirszfelda 12, 53-413 Wroclaw, Poland; pawel.lesiak@dcopih.pl; 5 Department of Oncological Surgery, Gdynia Oncology Centre, 81-519 Gdynia, Poland; maccies@gumed.edu.pl (M.C.); piotr_kurek@gumed.edu.pl (P.K.); 6 Department of Oncological Surgery, Faculty of Health Sciences with the Institute of Maritime and Tropical Medicine, Medical University of Gdańsk, 81-519 Gdynia, Poland; 7 Department of General and Oncological Surgery, Voivodeship Hospital in Bialystok, 15-000 Bialystok, Poland; marcinj74@gmail.com (M.J.); damianboxlek@gmail.com (D.N.); 8 Department of Medicine, Academy of Applied Medical and Social Sciences-AMiSNS (Akademia Medycznych I Spolecznych Nauk Stosowanych), AMiSNS Ul. Lotnicza 3, 82-300 Elbląg, Poland

**Keywords:** colorectal cancer, anastomotic leak, postoperative complications, indocyanine green, fluorescence angiography, colorectal surgery, surgical quality

## Abstract

**Background:** Anastomotic leak remains a major complication after colorectal cancer surgery. While most studies focus on prediction or prevention, less is known about the clinical course after leak diagnosis. We aimed to evaluate the occurrence, severity, management, and early postoperative consequences of anastomotic leak after elective colorectal cancer surgery. **Methods:** This retrospective multicenter cohort study included consecutive patients undergoing elective colorectal cancer resection with restoration of bowel continuity in five tertiary referral centers. Patients without an evaluable anastomosis were excluded. The primary endpoint was 30-day anastomotic leak incidence. Secondary endpoints included Clavien–Dindo severity, therapeutic management, major morbidity, surgical reintervention, length of hospital stay, intensive care unit admission, readmission, and 30-day mortality. Firth penalized logistic regression was used for adjusted analyses. **Results:** Among 943 patients with an evaluable anastomosis, 55 developed an anastomotic leak (5.8%). Leak was more frequent after rectal than colon cancer surgery (8.8% vs. 4.5%; crude OR 2.04, 95% CI 1.18–3.53; *p* = 0.016). After adjustment, rectal cancer remained independently associated with leak occurrence (adjusted OR 3.61, 95% CI 1.74–7.48; *p* < 0.001), whereas intraoperative indocyanine green fluorescence angiography was not (adjusted OR 1.02, 95% CI 0.48–2.17; *p* = 0.962). Once leak occurred, 92.7% of patients developed major morbidity, 76.4% required surgical reintervention, and, among patients with available length-of-stay data, 65.4% had a postoperative hospital stay exceeding 14 days; 30-day mortality was 10.9%. **Conclusions:** Anastomotic leak occurred in fewer than 6% of elective colorectal cancer resections but was associated with severe postoperative deterioration. Its impact is better captured by evaluating severity, management, and downstream consequences, rather than incidence alone.

## 1. Introduction

Anastomotic leak remains one of the most feared complications following colorectal cancer surgery [1]. Despite advances in surgical technique, perioperative care, and enhanced recovery pathways, clinically relevant leaks continue to occur in a meaningful proportion of patients and are consistently associated with postoperative morbidity, prolonged hospitalization, reintervention, impaired recovery, and early mortality [2]. Beyond the immediate postoperative period, anastomotic leak may also delay adjuvant treatment, increase healthcare costs, impair quality of life, and adversely influence long-term oncological outcomes [3,4,5].

Most previous research has focused on identifying patients at increased risk of anastomotic failure or on evaluating strategies to reduce its occurrence [6]. Patient-related factors, tumor location, operative complexity, anastomotic configuration, diverting stoma formation, and perioperative optimization have all been extensively investigated [7]. More recently, intraoperative assessment of bowel perfusion with indocyanine green (ICG) fluorescence angiography has received considerable attention. Randomized trials and meta-analyses suggest that fluorescence-guided perfusion assessment may reduce leak rates in selected settings, although observational cohorts remain difficult to interpret because ICG is often used preferentially in technically demanding procedures [8,9,10,11,12].

Although prediction and prevention remain clinically important, they capture only part of the problem. In many studies, anastomotic leak is treated as a binary postoperative endpoint: present or absent [12]. This approach is useful for benchmarking incidence, but it does not describe what happens after the diagnosis is made. In routine practice, a leak may range from a contained collection managed conservatively to a life-threatening septic complication requiring repeated interventions, intensive care support, and prolonged hospitalization [13]. Patients classified under the same endpoint may therefore experience markedly different clinical courses.

This distinction is relevant for surgical quality assessment. Contemporary outcome evaluation increasingly recognizes that postoperative quality depends not only on whether complications occur, but also on how severe they are, how rapidly they are recognized, and how effectively they are managed [14]. Concepts such as failure-to-rescue and composite quality indicators, including Textbook Outcome, have reinforced the importance of looking beyond isolated complication rates [15,16,17,18]. In this context, anastomotic leak may be better understood as a multidimensional postoperative event involving occurrence, severity, therapeutic escalation, and downstream consequences [17,18,19,20].

**Figure 1:**
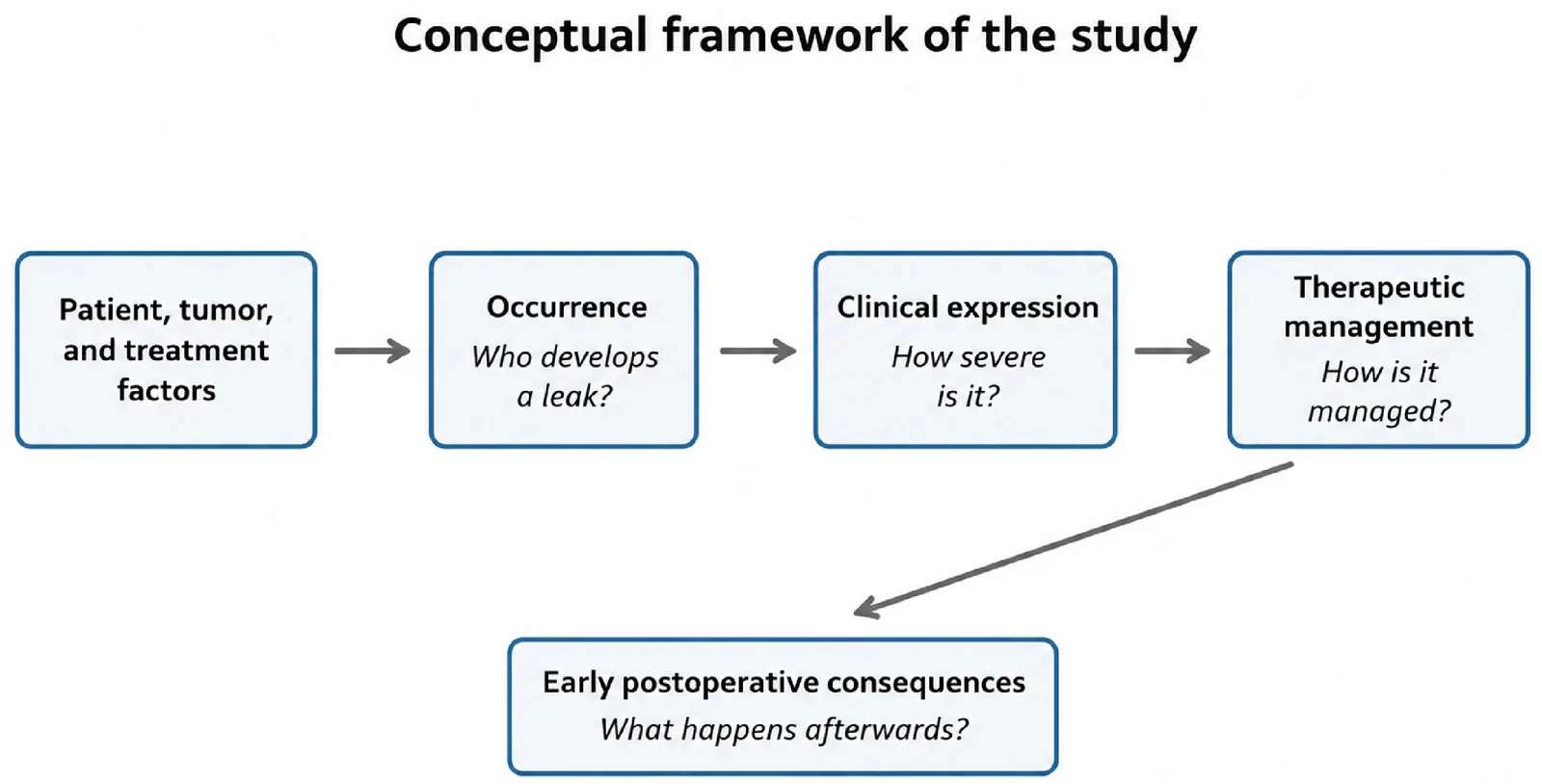
Conceptual framework of the study. The study evaluated anastomotic leak as a multidimensional postoperative event, integrating patient, tumor, and treatment factors with leak occurrence, clinical expression, therapeutic management, and downstream consequences.

The present multicenter cohort study was designed to characterize this broader clinical trajectory after elective colorectal cancer surgery. Rather than focusing exclusively on leak prediction, we evaluated four predefined domains: leak occurrence, clinical expression, therapeutic management, and early postoperative consequences. As a secondary objective, we explored contemporary patterns of ICG fluorescence angiography use and assessed its adjusted association with anastomotic leak, acknowledging the inherent limitations of observational data for causal inference (Figure 1).

## 2. Materials and Methods

### 2.1. Study Design and Setting

This was a retrospective multicenter cohort study based on the secondary analysis of data prospectively entered into institutional clinical databases as part of routine clinical practice. The study included consecutive patients undergoing elective colorectal cancer resection at five tertiary referral centers in Poland between January 2022 and August 2025. The research question, eligibility criteria, outcomes, domain-based analytical framework, and statistical analysis plan were prespecified before construction of the final analytical dataset and statistical analysis. No study-specific prospective enrollment or intervention was undertaken. Four complementary domains were evaluated: leak occurrence, clinical expression, therapeutic management, and early postoperative consequences. The study was conducted in accordance with the Declaration of Helsinki and is reported following the Strengthening the Reporting of Observational Studies in Epidemiology (STROBE) recommendations [21] (Supplementary Materials). Ethical approval was obtained from the Ethics Committee of the Academy of Applied Medical and Social Sciences, Elbląg, Poland (approval No. KB10/2024; 10 July 2024). Owing to the retrospective design and complete anonymization of the dataset, the requirement for individual informed consent was waived.

### 2.2. Study Population

The source database included 1124 consecutive patients undergoing elective surgery for histologically confirmed colorectal adenocarcinoma. For the present analysis, only patients who underwent bowel reconstruction with restoration of intestinal continuity were considered eligible. Patients undergoing Hartmann’s procedure, permanent end colostomy, or any procedure without a postoperative evaluable anastomosis were excluded because the primary endpoint could not occur. Patients with missing information regarding the occurrence of anastomotic leak were also excluded.

**Figure 2:**
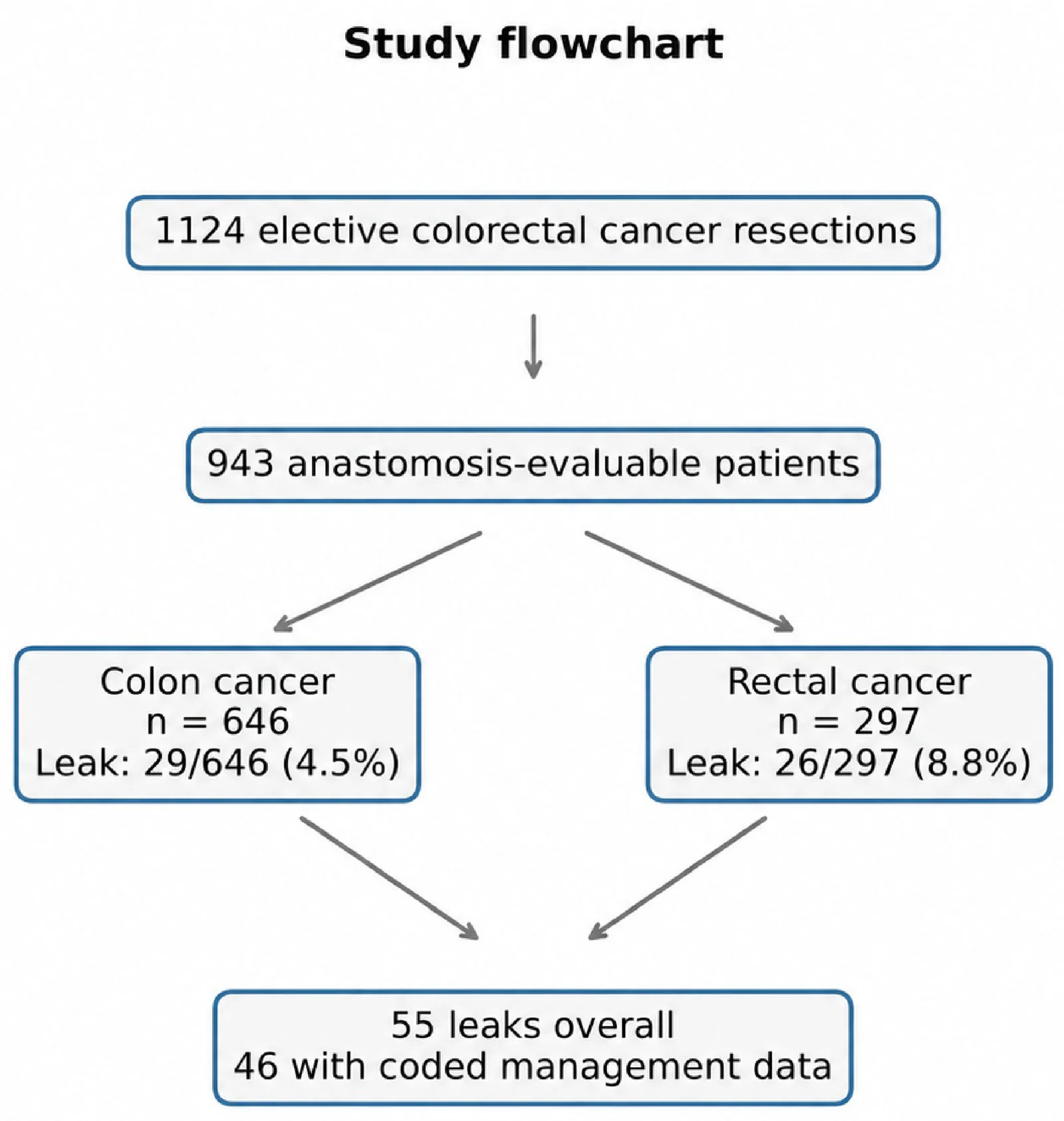
Study flowchart. Flow diagram showing the source cohort, anastomosis-evaluable population, tumor-location subgroups, leak occurrence, and management-coded leak cohort.

The final study cohort consisted of 943 patients with an evaluable intestinal anastomosis (Figure 2). Tumors were categorized according to anatomical location as colon or rectal cancers, and analyses were prespecified to consider these groups separately whenever clinically appropriate.

### 2.3. Data Collection and Operative Characteristics

Clinical data were extracted from institutional prospectively maintained databases using standardized definitions shared among participating centers. Baseline variables included age, sex, body mass index (BMI), American Society of Anesthesiologists (ASA) physical status, age-adjusted Charlson Comorbidity Index (aCCI), tumor location, neoadjuvant treatment, operative approach, intraoperative use of ICG fluorescence angiography, and participating center. Neoadjuvant treatment was defined as any preoperative chemotherapy, radiotherapy, chemoradiotherapy, or combined oncological treatment administered before definitive surgical resection. Anastomotic technique was extracted from the operative records and harmonized across participating centers into four categories: linear stapled, circular stapled, completely hand-sewn, and hybrid stapled/hand-sewn. The hybrid category included anastomoses constructed using a combination of stapling devices and manual suturing. Linear staplers were used predominantly for ileocolic and colocolic reconstructions, whereas circular staplers were used predominantly for colorectal and coloanal anastomoses. All colorectal and coloanal anastomoses underwent routine intraoperative integrity testing using an air leak test, commonly referred to as the “bubble test.” The pelvis was filled with sterile saline, and air was insufflated transanally; the appearance of air bubbles was considered indicative of an anastomotic defect. When the initial test was positive, the anastomosis was reinforced or reconstructed, as clinically appropriate, and the test was repeated. A proximal diverting loop ileostomy could additionally be created at the operating surgeon’s discretion, particularly in the presence of a low anastomosis, an initially positive leak test, or other intraoperative concerns regarding anastomotic integrity.

For patients undergoing rectal cancer resection with restoration of bowel continuity, planned covering loop ileostomy status was retrospectively verified through review of the operative reports. A planned covering ileostomy was defined as a temporary diverting loop ileostomy created during the index operation as part of the intended operative strategy, while the colorectal or coloanal anastomosis was retained. Stomas created unexpectedly during the index operation or during postoperative reintervention were not classified as planned covering ileostomies and were analyzed separately as unplanned diversion or rescue procedures, respectively. An unplanned diverting stoma was defined as a temporary fecal diversion, either a loop ileostomy or loop colostomy, created during the index operation despite not being included in the preoperative surgical plan, while an evaluable anastomosis was retained. This category did not include planned covering ileostomies, Hartmann procedures, permanent end colostomies, or stomas created during postoperative reintervention. All patients received intravenous antimicrobial prophylaxis according to the institutional protocols adopted at the participating centers. The standard regimen used across the participating network consisted of cefazolin 2 g combined with metronidazole 500 mg, administered within 60 min before surgical incision. Intraoperative redosing of cefazolin was performed during prolonged procedures when clinically indicated [22]. Selective cyclooxygenase-2 (COX-2) inhibitors were not administered during the perioperative period to any patient included in the study cohort. Postoperative biochemical testing was performed according to local clinical practice and individual patient requirements. However, serial values of C-reactive protein, leukocyte count, procalcitonin, and other inflammatory biomarkers were not included in the shared multicenter database. Consequently, no standardized biochemical screening protocol or biomarker-based diagnostic analysis could be evaluated in the present study.

### 2.4. Outcomes

The primary outcome was the occurrence of anastomotic leak within 30 days after surgery. Anastomotic leak was defined according to the International Study Group of Rectal Cancer as a defect of the intestinal wall at the anastomotic site resulting in communication between the intra- and extraluminal compartments, confirmed clinically, radiologically, endoscopically, or during reoperation [3].

Leak-specific severity was graded according to the International Study Group of Rectal Cancer (ISREC) classification [23]. Grade A leakage required no change in postoperative management; grade B required active therapeutic intervention without relaparotomy, including systemic antibiotic therapy, radiologically guided drainage, or endoscopic treatment; and grade C required relaparotomy. An ISREC grade was assigned only to patients for whom the leak-management pathway was available.

The Clavien–Dindo classification was applied separately to characterize the severity of the overall postoperative course, including complications and clinical consequences not necessarily attributable solely to the anastomotic leak [24]. For descriptive purposes, the overall postoperative course was grouped into four clinically meaningful categories: Clavien–Dindo grade <III, grade IIIa, grades IIIb–IV, and grade V. Major postoperative morbidity was defined as Clavien–Dindo grade III or higher. Thus, ISREC grading reflected the treatment required specifically for anastomotic leakage, whereas Clavien–Dindo grading captured the overall therapeutic burden experienced by the patient.

Secondary outcomes included leak-specific ISREC grade, Clavien–Dindo severity, therapeutic management, major postoperative morbidity, surgical reintervention, prolonged postoperative hospital stay (>14 days), unplanned intensive care unit (ICU) admission, 30-day readmission, and 30-day mortality. Prolonged postoperative hospital stay was defined as a postoperative length of stay exceeding 14 days. This outcome was evaluated only in patients with an available postoperative length-of-stay value. Postoperative surveillance for anastomotic leakage was based on routine clinical assessment during the index hospitalization and after discharge within 30 days. Additional radiological, endoscopic, or operative evaluation was performed when leakage was clinically suspected, according to local institutional practice. Routine computed tomography screening was not performed in asymptomatic patients. Surgical reintervention was defined as any postoperative surgical procedure or return to the operating room recorded during the postoperative course.

### 2.5. Therapeutic Management

Management of anastomotic leak was categorized according to the highest level of treatment required: conservative management, radiological intervention, endoscopic treatment, surgical treatment, or combined endoscopic and surgical treatment. Conservative management was defined as systemic antibiotic therapy without radiological, endoscopic, or surgical intervention. Therapeutic antibiotic selection following leak diagnosis was guided by clinical severity, microbiological findings, renal function, local antimicrobial susceptibility patterns, and institutional antimicrobial stewardship protocols. Patients with documented anastomotic leak but missing or non-coded treatment information were retained in all analyses evaluating leak occurrence, severity, predictors, and postoperative outcomes. They were excluded only from analyses specifically describing therapeutic management.

### 2.6. ICG Fluorescence Angiography

Because the use of ICG fluorescence angiography was left to surgeon preference and institutional practice rather than determined by a standardized protocol, fluorescence imaging was considered a contextual intraoperative variable rather than an intervention under investigation. Its use was expected to be influenced by operative complexity, tumor location, surgical approach, and neoadjuvant treatment. Consequently, analyses involving ICG were regarded as exploratory and interpreted as describing contemporary patterns of clinical implementation rather than causal treatment effects [8].

### 2.7. Statistical Analysis

Continuous variables are presented as median and interquartile range (IQR), whereas categorical variables are summarized as frequencies and percentages. Comparisons between groups were performed using the Mann–Whitney U test for continuous variables and Pearson’s chi-square test or Fisher’s exact test for categorical variables, as appropriate.

The primary multivariable analysis evaluated independent determinants of anastomotic leak using Firth penalized logistic regression, selected a priori because of the relatively small number of leak events and its ability to reduce small-sample estimation bias and separation [25,26]. Covariates were selected before analysis according to clinical relevance and evidence from the literature rather than automated statistical procedures. The primary model included age, BMI, ASA class (≥III), tumor location, neoadjuvant treatment, operative approach, ICG fluorescence angiography, and participating center, which was entered as a fixed adjustment factor.

To evaluate the independent clinical consequences of anastomotic leak, additional multivariable Firth logistic regression models were constructed using major postoperative morbidity, surgical reintervention, prolonged hospital stay, ICU admission, 30-day readmission, and 30-day mortality as dependent variables. These models were adjusted for age, BMI, ASA class (≥III), tumor location, neoadjuvant treatment, operative approach, and participating center when event counts allowed; the mortality model was reduced because of the limited number of deaths.

Missingness for variables used in the main multivariable analyses was low for most covariates, including BMI (2.4%) and ASA class (1.5%). ICG fluorescence angiography status was unavailable for one patient (0.1%). This record was treated as missing rather than interpreted as absence of ICG use. The patient was retained in analyses not involving ICG but excluded from ICG-specific comparisons and multivariable models including ICG as a covariate. ICG-related percentages were calculated using available-case denominators, and no imputation was performed. For binary postoperative event variables, the absence of a positive event code was interpreted as no recorded event after internal consistency checks. Missing continuous postoperative length-of-stay values were not interpreted as absence of prolonged hospitalization. Patients with missing length-of-stay data were excluded only from LOS-specific descriptive and multivariable analyses and remained eligible for all other analyses. Complete-case analyses were performed without multiple imputation. Patients with missing treatment information remained included in all analyses except those specifically describing management strategies.

Model discrimination was evaluated using the area under the receiver operating characteristic curve (AUC). Internal validation was performed using bootstrap resampling with 1000 iterations, and overall model performance was additionally assessed using the Brier score and calibration slope. All statistical tests were two-sided, and a *p* value < 0.05 was considered statistically significant. Statistical analyses were performed using R version 4.5.3 (R Foundation for Statistical Computing, Vienna, Austria).

## 3. Results

### 3.1. Study Cohort

Among 1124 consecutive patients who underwent elective colorectal cancer resection, 943 met the eligibility criteria and underwent bowel reconstruction with an evaluable intestinal anastomosis. Of these, 646 patients (68.5%) underwent colon cancer resection and 297 (31.5%) underwent rectal cancer surgery. ICG fluorescence angiography status was available for 942 of 943 patients. ICG was used in 540 patients (57.3%) and was not used in 402 patients (42.7%); ICG status was unavailable for one patient. Among the 943 evaluable anastomoses, 846 (89.7%) were constructed entirely using stapling devices, including 318 linear-stapled anastomoses (33.7%) and 528 circular-stapled anastomoses (56.0%). Ninety-six anastomoses (10.2%) were completely hand-sewn, whereas one anastomosis (0.1%) was constructed using a hybrid stapled and hand-sewn technique. Following retrospective verification of the operative reports, a planned covering loop ileostomy was documented in 49 of the 297 patients undergoing rectal cancer resection with restoration of bowel continuity (16.5%). Baseline characteristics according to tumor location and leak status are summarized in Table 1 and Table 2.

### 3.2. Occurrence of Anastomotic Leak

**Figure 3:**
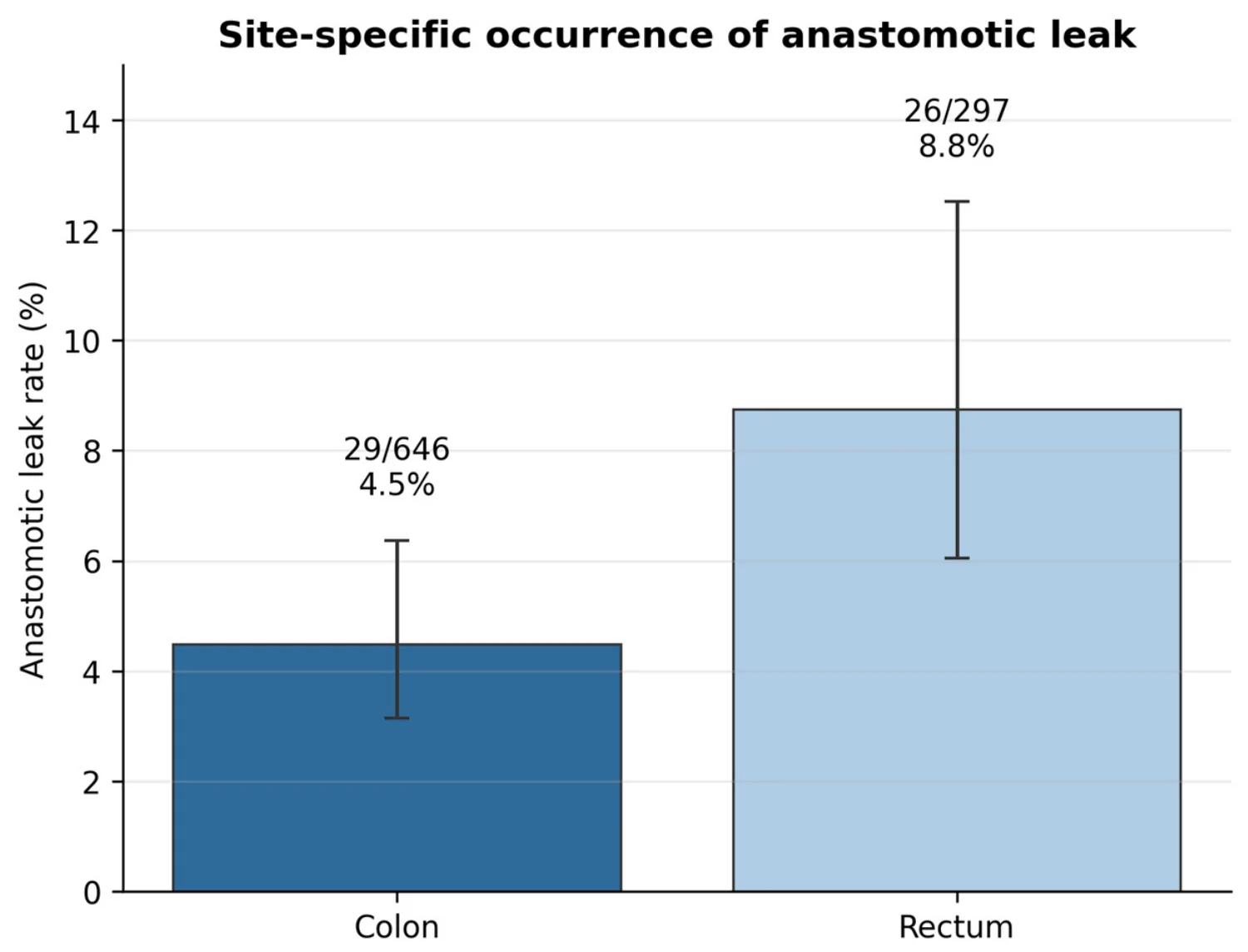
Site-specific occurrence of anastomotic leak. Bar plot showing leak rates after colon and rectal cancer surgery. Error bars indicate Wilson 95% confidence intervals.

Overall, anastomotic leak occurred in 55 of 943 patients, corresponding to an incidence of 5.8%. Leak occurrence differed according to tumor location. Among patients undergoing colon cancer resection, 29 of 646 patients developed a leak (4.5%). In contrast, leak occurred in 26 of 297 patients undergoing rectal cancer surgery (8.8%), corresponding to an absolute risk difference of 4.3 percentage points and an approximately two-fold increase in crude risk (OR 2.04, 95% CI 1.18–3.53; *p* = 0.016) (Figure 3).

**Figure 4:**
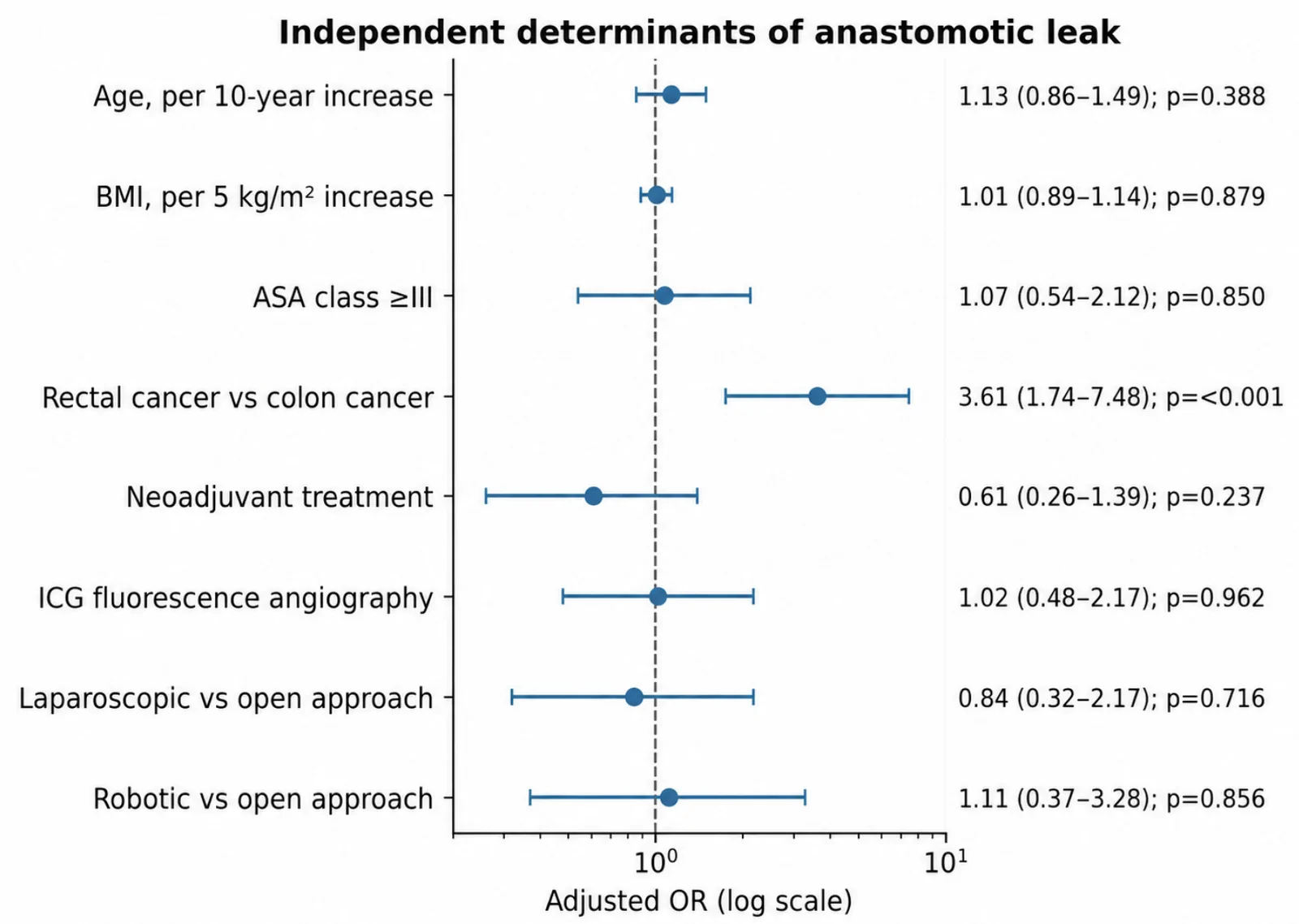
Independent determinants of anastomotic leak. Forest plot showing adjusted odds ratios from the primary Firth penalized logistic regression model. The vertical dashed line indicates no association.

In the primary Firth penalized multivariable model, rectal cancer remained the dominant independent determinant of leak (adjusted OR 3.61, 95% CI 1.74–7.48; *p* < 0.001). Age, BMI, ASA class ≥ III, neoadjuvant treatment, operative approach, and ICG use were not independently associated with leak occurrence. Model discrimination was acceptable, with an apparent AUC of 0.724 and a bootstrap median AUC of 0.691. The Brier score was 0.052, and the bootstrap median calibration slope was 0.727 (Table 3 and Table 4, Figure 4).

### 3.3. Clinical Expression of Anastomotic Leak

Leak-specific grading according to the ISREC classification was possible for 46 of the 55 patients with anastomotic leak for whom management data were available. No patient was classified as ISREC grade A. Twelve patients (26.1%) had grade B leakage requiring active nonoperative treatment, whereas 34 (73.9%) had grade C leakage requiring surgical reintervention. Nine patients could not be assigned an ISREC grade because their leak-management pathway was missing or not coded.

The Clavien–Dindo classification was applied separately to characterize the severity of the overall postoperative course. Among all 55 patients who developed an anastomotic leak, four (7.3%) experienced complications below grade III and three (5.5%) were classified as grade IIIa. Severe postoperative courses predominated: 42 patients (76.4%) developed grade IIIb–IV complications, and six (10.9%) died within 30 days, corresponding to grade V. Overall, 51 of 55 patients with leakage (92.7%) fulfilled the definition of major postoperative morbidity (Table 5).

### 3.4. Therapeutic Management of Anastomotic Leak

**Figure 5:**
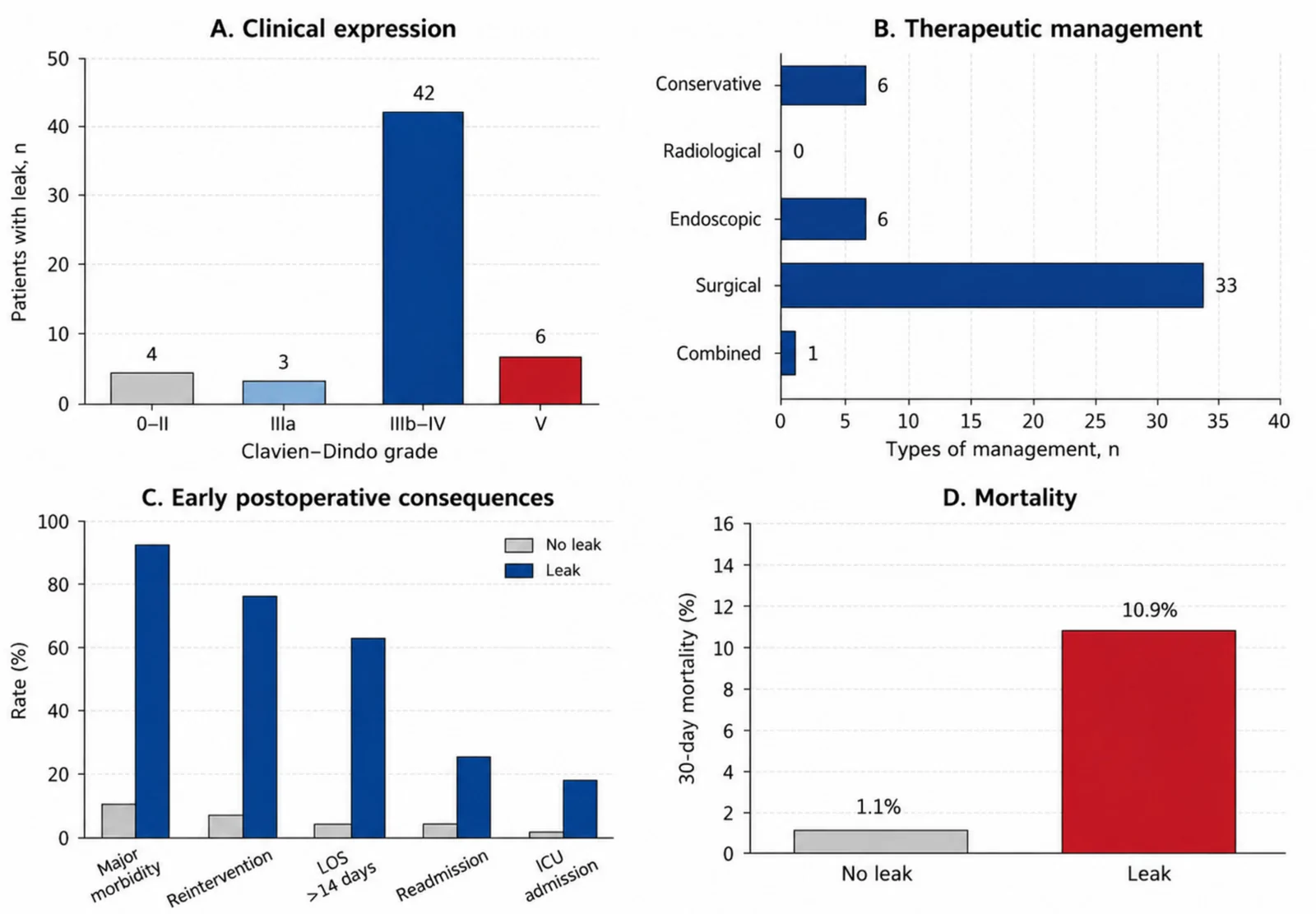
Multidimensional clinical trajectory of anastomotic leak. (**A**) shows clinical expression according to Clavien–Dindo category. (**B**) shows leak-specific therapeutic management among patients with coded treatment data. (**C**) shows early postoperative consequences in patients with and without leak. (**D**) shows 30-day mortality according to leak status.

Management data were available for 46 of the 55 patients with anastomotic leak. Nine patients with missing or non-coded treatment information were excluded from the management-specific analysis but retained in all other analyses. Among patients with documented management pathways, surgical treatment represented the predominant leak-specific rescue strategy and was required in 33 of 46 patients (71.7%). Six patients (13.0%) were successfully managed with systemic antibiotic therapy alone, without radiological, endoscopic, or surgical intervention. Endoscopic treatment alone was performed in six (13.0%), and one patient (2.2%) required combined endoscopic and surgical management. No patient underwent exclusively radiological treatment as the definitive management strategy (Table 6 and Figure 5).

### 3.5. Early Postoperative Consequences of Anastomotic Leak

Anastomotic leak was associated with marked deterioration in early postoperative recovery. Major postoperative morbidity occurred in 51 of 55 patients with leak (92.7%) compared with 74 of 888 patients without leak (8.3%), corresponding to an absolute risk difference of 84.4 percentage points. Surgical reintervention was required in 42 of 55 patients with leak (76.4%) compared with 49 of 888 patients without leak (5.5%). Postoperative length-of-stay data were available for 934 patients, including 52 patients with anastomotic leak and 882 without leak. A postoperative stay exceeding 14 days occurred in 34 of 52 patients with leak (65.4%) compared with 28 of 882 patients without leak (3.2%), corresponding to an absolute risk difference of 62.2 percentage points. Readmission within 30 days occurred in 14 of 55 patients with leak (25.5%) compared with 26 of 888 without leak (2.9%), while unplanned ICU admission occurred in 10 of 55 (18.2%) and 12 of 888 (1.4%), respectively. Thirty-day mortality was 10.9% among patients with leak and 1.1% among those without leak.

**Figure 6:**
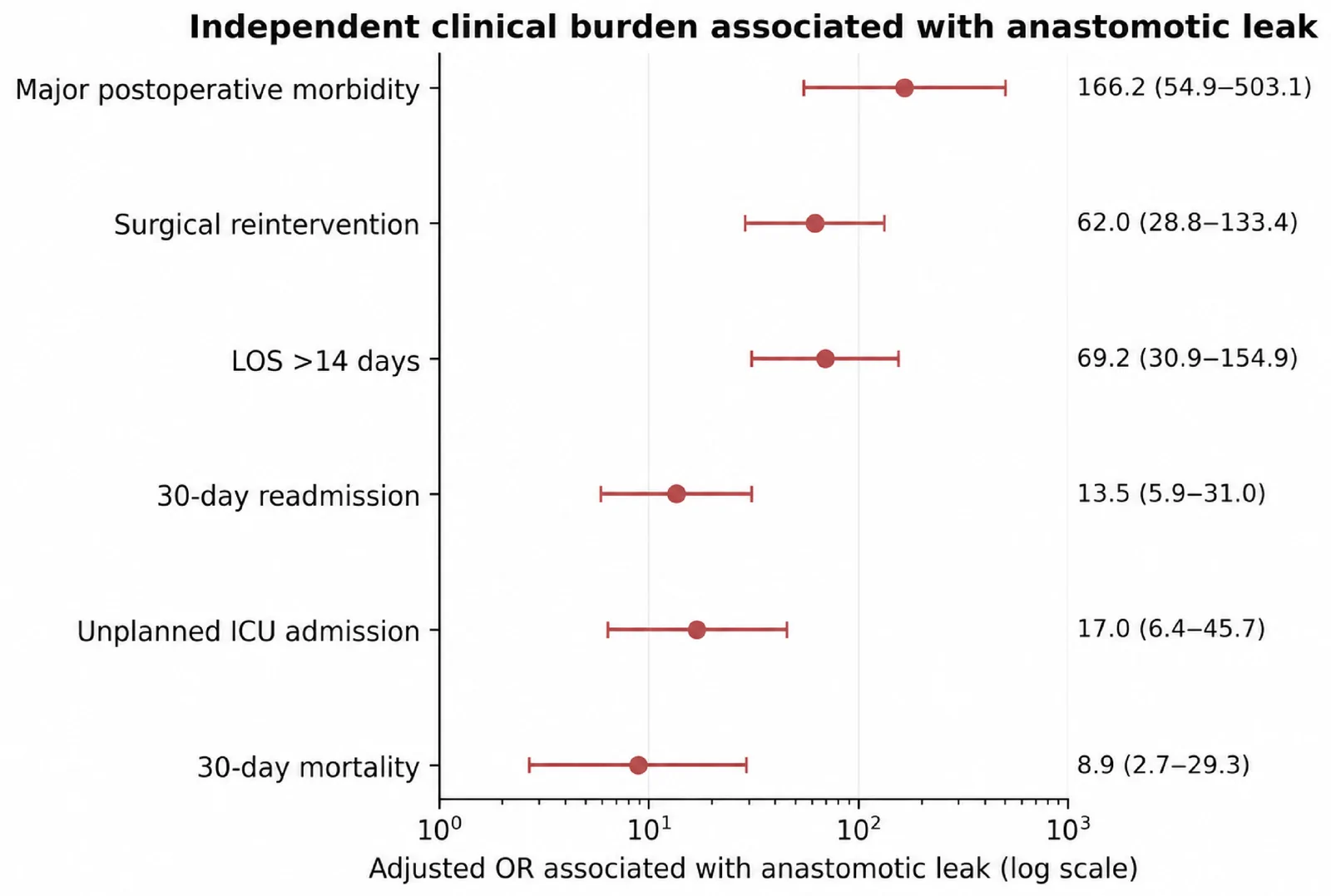
Independent clinical burden associated with anastomotic leak. Forest plot showing adjusted associations between anastomotic leak and postoperative outcomes. These estimates describe clinical association and should not be interpreted as causal effects.

After adjustment for baseline clinical and operative factors, anastomotic leak remained independently associated with major morbidity, surgical reintervention, prolonged hospitalization, 30-day readmission, ICU admission, and 30-day mortality. The magnitude of these associations is shown in Table 7 and Figure 6.

### 3.6. Contemporary Use of ICG Fluorescence Angiography

Patterns of ICG fluorescence angiography suggested selective adoption according to case complexity and operative context. ICG was used in 240 of 297 rectal resections (80.8%) compared with 300 of 645 colon resections (46.5%). It was also used in 266 of 309 robotic procedures (86.1%), 236 of 365 laparoscopic procedures (64.7%), and 38 of 268 open operations (14.2%). Among patients receiving neoadjuvant treatment, ICG was used in 148 of 194 cases (76.3%), compared with 392 of 748 patients who did not receive neoadjuvant treatment (52.4%) (Table 8).

Among the 942 patients with available ICG status, leak occurred in 35 of 540 patients treated with ICG (6.5%) and in 20 of 402 patients treated without ICG (5.0%) (crude OR 1.32, 95% CI 0.75–2.33; *p* = 0.400). After multivariable adjustment, ICG fluorescence angiography was not independently associated with leak occurrence (adjusted OR 1.02, 95% CI 0.48–2.17; *p* = 0.962).

## 4. Discussion

This multicenter cohort study provides an integrated characterization of anastomotic leak after elective colorectal cancer surgery by evaluating occurrence, clinical expression, therapeutic management, and early postoperative consequences within a single analytical framework. Three observations are particularly relevant. First, although leak occurred in fewer than 6% of patients, it was associated with substantial postoperative deterioration, including markedly increased major morbidity, reintervention, prolonged hospitalization, intensive care utilization, readmission, and early mortality. Second, rectal cancer remained the strongest independent determinant of leak occurrence after adjustment for patient- and procedure-related factors. Third, the apparent association between ICG fluorescence angiography and leak disappeared after adjustment, suggesting that fluorescence imaging in this cohort largely reflected selective use in more complex cases rather than a directly measurable protective effect.

These findings support the view that the clinical importance of anastomotic leak cannot be adequately captured by incidence alone [27]. Many studies report leak as a binary quality indicator and focus primarily on prevention or prediction [12]. While this remains important, it does not fully describe what happens once a leak occurs. In the present cohort, more than 90% of patients with leak developed major postoperative morbidity, approximately three-quarters required operative rescue, nearly two-thirds experienced prolonged hospitalization, and approximately one in ten died within 30 days. This pattern suggests that anastomotic leak functions less as an isolated complication than as the beginning of a cascade of postoperative deterioration [19,28].

The higher leak rate observed after rectal cancer surgery is consistent with previous literature and likely reflects the anatomical and technical complexity of pelvic dissection, limited operative exposure, anastomotic tension, and potentially impaired tissue healing after neoadjuvant treatment [4]. Notably, the adjusted association between rectal cancer and leak was stronger than the crude association. This may reflect negative confounding by factors such as younger age and lower ASA class among patients with rectal cancer, which partially masked the underlying procedure-related risk in unadjusted comparisons.

A relevant strength of the present analysis is the distinction between leak occurrence and leak severity. Mild presentations were uncommon, whereas severe complications predominated. More than three-quarters of patients experienced Clavien–Dindo grade IIIb-IV complications, and postoperative mortality approached 11%. This finding is clinically important because two patients classified as having an anastomotic leak may require very different treatment pathways and experience very different outcomes [3].

The management data further reinforce this interpretation. Among patients with coded treatment pathways, nearly three-quarters required surgical treatment for leak control, whereas conservative or endoscopic management alone was successful in a minority. This does not imply that surgery should be the default treatment for every leak; rather, it reflects the case mix of clinically recognized leaks in a tertiary multicenter cohort and the severity of postoperative deterioration observed in many patients. The discrepancy between leak-specific surgical management and the broader surgical reintervention endpoint should be interpreted in this context, as the latter captures any postoperative surgical reintervention during the index episode.

Early recognition remains a critical component of anastomotic leak management, particularly because clinical manifestations may initially be subtle or nonspecific. Serial postoperative C-reactive protein (CRP) measurements have emerged primarily as a means of identifying patients at low risk of clinically relevant leakage rather than as a stand-alone diagnostic test. In the prospective multicenter PREDICT study, an increase in CRP exceeding 50 mg/L between consecutive postoperative measurements showed a sensitivity of 85% and a negative predictive value of 99% for anastomotic leak requiring intervention; changes occurring between postoperative days 3 and 5 also demonstrated high specificity [29]. Similarly, Messias et al. reported that a CRP concentration below 180 mg/L on postoperative day 4 had a negative predictive value of 97.2%, supporting its potential role in informing safe discharge pathways [30]. More recently, Bintintan et al. emphasized the importance of CRP kinetics rather than isolated measurements: a sustained decline after postoperative day 3 appeared to make leakage unlikely, whereas persistently elevated or only minimally decreasing values on postoperative days 5 and 7 warranted further clinical investigation [31]. Collectively, these findings suggest that dynamic CRP assessment may complement clinical surveillance and guide selective imaging. However, biomarker values should be interpreted within the broader clinical context and should not replace radiological, endoscopic, or operative assessment when leakage is suspected. Serial postoperative CRP and other inflammatory biomarkers were not captured in the present multicenter database and could therefore not be incorporated into our diagnostic or predictive analyses.

The role of ICG fluorescence angiography deserves cautious interpretation. Randomized trials and meta-analyses suggest that fluorescence-guided assessment of bowel perfusion may reduce leak rates in selected patients [8,9,10]. In our real-world cohort, however, ICG was preferentially used during rectal, minimally invasive, robotic, and neoadjuvant-treated cases. The absence of an independent association between ICG and leak should therefore not be interpreted as evidence against fluorescence-guided surgery. More appropriately, it illustrates the limitations of retrospective observational data for estimating the causal effect of a selectively adopted technology.

Some adjusted odds ratios describing the downstream consequences of leak were large. These effect sizes should not be interpreted as causal estimates in the strict sense; rather, they reflect the close biological and clinical relationship between anastomotic failure and subsequent postoperative deterioration. For this reason, absolute risk differences were reported alongside relative measures, as they may be more meaningful for clinicians and quality-improvement teams [32].

Several limitations should be acknowledged. First, the retrospective observational design introduces potential residual confounding despite multivariable adjustment. Second, some variables that may influence leak development, including anastomotic height, anastomotic configuration, vascular ligation level, timing of leak diagnosis, surgeon experience, detailed total mesorectal excision quality, and standardized perfusion quantification, were not uniformly available. Quantitative estimated intraoperative blood loss was also not uniformly captured in the shared multicenter database and could therefore not be included in the present analyses. Additionally, serial postoperative inflammatory biomarkers, including CRP, leukocyte count, and procalcitonin, were not captured in the shared database. We were therefore unable to evaluate their temporal trajectories or diagnostic performance for the early identification or exclusion of anastomotic leakage. Third, leak-specific severity was graded according to the ISREC classification only when management data were available; consequently, nine patients with missing or non-coded treatment pathways could not be assigned an ISREC grade. The Clavien–Dindo classification was retained in parallel because it captures the severity of the entire postoperative course rather than the leak-specific treatment requirement alone [23]. Fourth, management information was unavailable for nine patients with leak. Fifth, participating centers may have differed in perioperative pathways, thresholds for imaging, ICU admission, and reintervention, and this inter-center heterogeneity may have influenced downstream outcomes despite statistical adjustment for center. Finally, the present analysis focused on short-term outcomes; long-term oncological outcomes, permanent stoma formation, bowel function, and quality of life require dedicated future evaluation.

From a clinical perspective, these findings suggest that postoperative quality assessment should include not only whether anastomotic leak occurred, but also how severe it was, how it was managed, and what consequences followed. Future studies should integrate standardized leak definitions, time-to-diagnosis, rescue pathways, patient-centered recovery measures, and long-term oncological outcomes. Such an approach may better identify modifiable elements of postoperative care that remain hidden when leak is reported only as present or absent.

## 5. Conclusions

In this multicenter cohort of patients undergoing elective colorectal cancer surgery, anastomotic leak occurred in fewer than 6% of cases but was associated with substantial postoperative deterioration. Rectal cancer remained the principal independent determinant of leak occurrence, whereas ICG fluorescence angiography was not independently associated with leak after adjustment, likely reflecting selective use in more complex cases. These findings suggest that the clinical importance of anastomotic leak lies not only in its incidence but also in its severity, management, and downstream consequences. Quality-improvement initiatives should therefore address both leak prevention and standardized rescue pathways aimed at minimizing its clinical burden.

## Figures and Tables

**Table 1:** Baseline characteristics according to tumor location.

Variable	Overall (*n* = 943)	Colon (*n* = 646)	Rectum (*n* = 297)	*p* Value
Age, years	71.0 (63.0–76.0)	72.0 (65.0–77.0)	67.0 (60.0–73.0)	<0.001
Male sex	512/943 (54.3)	329/646 (50.9)	183/297 (61.6)	0.002
BMI, kg/m^2^	26.7 (23.9–29.8)	26.7 (24.0–30.0)	26.7 (23.9–29.7)	0.528
ASA class ≥ III	271/943 (28.7)	217/646 (33.6)	54/297 (18.2)	<0.001
aCCI	5.0 (4.0–6.0)	5.0 (4.0–6.0)	5.0 (4.0–5.0)	<0.001
Neoadjuvant treatment	194/943 (20.6)	7/646 (1.1)	187/297 (63.0)	<0.001
ICG fluorescence angiography **^§^**	540/942 (57.3)	300/645 (46.5)	240/297 (80.8)	<0.001
Open approach	268/943 (28.4)	213/646 (33.0)	55/297 (18.5)	<0.001
Laparoscopic approach	366/943 (38.8)	233/646 (36.1)	133/297 (44.8)	0.012
Robotic approach	309/943 (32.8)	200/646 (31.0)	109/297 (36.7)	0.086
Unplanned diverting stoma at index surgery **^†^**	37/943 (3.9)	9/646 (1.4)	28/297 (9.4)	<0.001
Planned covering loop ileostomy *	-	-	49/297 (16.5)	-
Operative time, min	146.0 (110.0–205.0)	130.0 (100.0–187.8)	170.0 (135.0–265.0)	<0.001
Postoperative LOS, days	6.1 (4.2–8.0)	6.0 (4.0–8.0)	6.3 (5.0–8.0)	0.126
Anastomotic leak	55/943 (5.8)	29/646 (4.5)	26/297 (8.8)	0.016

Values are median (IQR) or n/N (%). aCCI, age-adjusted Charlson Comorbidity Index; ICG, indocyanine green; LOS, length of stay. ^§^ ICG percentages were calculated using available data; ICG status was unavailable for one patient in the colon subgroup. ^†^ An unplanned diverting stoma was defined as a temporary loop ileostomy or loop colostomy created during the index operation despite not being included in the preoperative surgical plan, with an evaluable anastomosis retained. This category excluded planned covering ileostomies, Hartmann procedures, permanent end colostomies, procedures without an evaluable anastomosis, and stomas created during postoperative reintervention. * Applicable only to patients undergoing rectal cancer resection with restoration of bowel continuity. Planned covering loop ileostomy status was retrospectively ascertained from the operative reports and analyzed separately from unplanned diversion.

**Table 2:** Baseline characteristics according to anastomotic leak status.

Variable	No Leak (*n* = 888)	Leak (*n* = 55)	*p* Value
Age, years	70.5 (63.0–76.0)	72.0 (64.5–77.0)	0.304
BMI, kg/m^2^	26.7 (23.9–29.8)	27.7 (24.3–30.0)	0.565
aCCI	5.0 (4.0–6.0)	5.0 (4.0–6.0)	0.648
Operative time, min	145.0 (107.0–200.0)	165.0 (133.5–221.5)	0.015
Postoperative LOS, days	6.0 (4.0–7.2)	17.5 (12.0–26.5)	<0.001
Male sex	481/888 (54.2)	31/55 (56.4)	0.782
ASA class ≥ III	255/888 (28.7)	16/55 (29.1)	1.000
Rectal cancer	271/888 (30.5)	26/55 (47.3)	0.016
Neoadjuvant treatment	181/888 (20.4)	13/55 (23.6)	0.606
ICG fluorescence angiography **^§^**	505/887 (56.9)	35/55 (63.6)	0.400
Unplanned diverting stoma at index surgery **^†^**	31/888 (3.5)	6/55 (10.9)	0.017
Open approach	257/888 (28.9)	11/55 (20.0)	0.168
Laparoscopic approach	348/888 (39.2)	18/55 (32.7)	0.393
Robotic approach	283/888 (31.9)	26/55 (47.3)	0.025

Values are median (IQR) or n/N (%). aCCI, age-adjusted Charlson Comorbidity Index; ICG, indocyanine green; LOS, length of stay. ^§^ ICG percentages were calculated using available data; ICG status was unavailable for one patient in the no-leak group. ^†^ An unplanned diverting stoma was defined as a temporary loop ileostomy or loop colostomy created during the index operation despite not being included in the preoperative surgical plan, with an evaluable anastomosis retained. This category excluded planned covering ileostomies, Hartmann procedures, permanent end colostomies, procedures without an evaluable anastomosis, and stomas created during postoperative reintervention.

**Table 3:** Site-specific occurrence of anastomotic leak.

Tumor Site	Total	Leak	No Leak	Leak Rate	Absolute Risk Difference	Crude OR (95% CI)	*p* Value
Colon	646	29	617	4.5%	Reference	Reference	—
Rectum	297	26	271	8.8%	+4.3 percentage points	2.04 (1.18–3.53)	0.016

Crude odds ratio compares rectal versus colon cancer.

**Table 4:** Primary Firth penalized model for anastomotic leak occurrence.

Variable	Adjusted OR	95% CI	*p* Value
Age, per 10-year increase	1.13	0.86–1.49	0.388
BMI, per 5 kg/m^2^ increase	1.01	0.89–1.14	0.879
ASA class ≥ III	1.07	0.54–2.12	0.850
Rectal cancer vs. colon cancer	3.61	1.74–7.48	<0.001
Neoadjuvant treatment	0.61	0.26–1.39	0.237
ICG fluorescence angiography	1.02	0.48–2.17	0.962
Laparoscopic vs. open approach	0.84	0.32–2.17	0.716
Robotic vs. open approach	1.11	0.37–3.28	0.856
Participating center	Included	—	—

Outcome: 30-day anastomotic leak. Center was included as a fixed adjustment variable but individual center estimates are not shown. Model performance: apparent AUC = 0.724; bootstrap median AUC = 0.691; Brier score = 0.052; bootstrap calibration slope = 0.727.

**Table 5:** Leak-specific and overall postoperative severity.

Severity Classification	Overall	Colon	Rectum
ISREC grade, management-coded leaks	*n* = 46	*n* = 26	*n* = 20
Grade A	0/46 (0.0)	0/26 (0.0)	0/20 (0.0)
Grade B	12/46 (26.1)	5/26 (19.2)	7/20 (35.0)
Grade C	34/46 (73.9)	21/26 (80.8)	13/20 (65.0)
Clavien–Dindo grade, all leaks	*n* = 55	*n* = 29	*n* = 26
Grade < III	4/55 (7.3)	4/29 (13.8)	0/26 (0.0)
Grade IIIa	3/55 (5.5)	1/29 (3.4)	2/26 (7.7)
Grades IIIb–IV	42/55 (76.4)	19/29 (65.5)	23/26 (88.5)
Grade V	6/55 (10.9)	5/29 (17.2)	1/26 (3.8)
Major morbidity	51/55 (92.7)	25/29 (86.2)	26/26 (100.0)

ISREC grading describes leak-specific severity according to the treatment required and was available for 46 patients with coded management data. Clavien–Dindo grading describes the severity of the overall postoperative course and was available for all 55 patients with anastomotic leak. Nine patients without coded management data could not be assigned an ISREC grade. ISREC, International Study Group of Rectal Cancer.

**Table 6:** Distribution of treatment strategies among patients with documented leak management.

Management Strategy	Overall Coded Leaks (*n* = 46)	Colon Coded Leaks (*n* = 26)	Rectal Coded Leaks (*n* = 20)
Conservative	6/46 (13.0)	4/26 (15.4)	2/20 (10.0)
Radiological	0/46 (0.0)	0/26 (0.0)	0/20 (0.0)
Endoscopic	6/46 (13.0)	1/26 (3.8)	5/20 (25.0)
Surgical	33/46 (71.7)	20/26 (76.9)	13/20 (65.0)
Combined endoscopic-surgical	1/46 (2.2)	1/26 (3.8)	0/20 (0.0)

Only patients with coded management data are included; nine leak patients with missing or non-coded treatment were excluded from this table only.

**Table 7:** Comparison of early postoperative outcomes between patients with and without anastomotic leak.

Outcome	Leak (*n* = 55)	No Leak (*n* = 888)	Absolute Risk Difference	Adjusted OR (95% CI)	Adjusted *p* Value
Major postoperative morbidity	51/55 (92.7)	74/888 (8.3)	+84.4 pp	166.2 (54.9–503.1)	<0.001
Surgical reintervention	42/55 (76.4)	49/888 (5.5)	+70.8 pp	62.0 (28.8–133.4)	<0.001
LOS > 14 days	34/52 (65.4)	28/882 (3.2)	+62.2 pp	69.2 (30.9–154.9)	<0.001
30-day readmission	14/55 (25.5)	26/888 (2.9)	+22.5 pp	13.5 (5.9–31.0)	<0.001
Unplanned ICU admission	10/55 (18.2)	12/888 (1.4)	+16.8 pp	17.0 (6.4–45.7)	<0.001
30-day mortality	6/55 (10.9)	10/888 (1.1)	+9.8 pp	8.9 (2.7–29.3)	<0.001

Adjusted models include age, BMI, ASA class, tumor location, neoadjuvant treatment, operative approach, and center; the mortality model was reduced because of limited events. pp, percentage points.

**Table 8:** Real-world selection of ICG fluorescence angiography.

Subgroup	Total	ICG Use	ICG Rate
Overall	942	540	57.3%
Colon cancer	645	300	46.5%
Rectal cancer	297	240	80.8%
Open surgery	268	38	14.2%
Laparoscopic surgery	365	236	64.7%
Robotic surgery	309	266	86.1%
Neoadjuvant treatment	194	148	76.3%
No neoadjuvant treatment	748	392	52.4%

ICG use is described as a contextual surgical variable and should not be interpreted causally.

## Data Availability

The datasets analyzed during the current study are not publicly available due to institutional and data-protection restrictions but may be available from the corresponding author upon reasonable request and subject to approval by the participating centers.

## Supplementary Materials

The following supporting information can be downloaded at: https://www.mdpi.com/article/10.3390/jcm15176903/s1, File: STROBE Statement—Checklist of items that should be included in reports of cohort studies.

## Abbreviations

The following abbreviations are used in this manuscript:

aCCI	Age-adjusted Charlson Comorbidity Index
ASA	American Society of Anesthesiologists
AUC	Area Under the Receiver Operating Characteristic Curve
BMI	Body Mass Index
CI	Confidence Interval
CT	Computed Tomography
ICG	Indocyanine Green
ICU	Intensive Care Unit
IQR	Interquartile Range
ISREC	International Study Group of Rectal Cancer
LOS	Length of Stay
OR	Odds Ratio
STROBE	Strengthening the Reporting of Observational Studies in Epidemiology
