# Supplementary material for: Clinical Burden of Anastomotic Leak After Elective Colorectal Cancer Surgery: A Multicenter Cohort Study
Source: J Clin Med. 2026 Sep 6;15(17):6903. doi: 10.3390/jcm15176903 (PMC13566583; doi:10.3390/jcm15176903)
Supplement: Supplementary file 1 [file jcm-15-06903-s001.zip › jcm-4444214-supplementary.pdf]

STROBE Statement—Checklist of items that should be included in reports of cohort studies

Manuscript: Clinical Burden of Anastomotic Leak After Elective Colorectal Cancer Surgery: A Multicenter Cohort Study

Section/topic	Item No	Recommendation	Reported on page/section
Title and abstract	1(a)	Indicate the study's design with a commonly used term in the title or the abstract	Page 1; Title page; Abstract - Methods
Title and abstract	1(b)	Provide in the abstract an informative and balanced summary of what was done and what was found	Page 2; Abstract - Background, Methods, Results, Conclusions
**Introduction**			
Background/rationale	2	Explain the scientific background and rationale for the investigation being reported	Pages 2-3; Introduction - paragraphs 1-4
Objectives	3	State specific objectives, including any prespecified hypotheses	Page 3; Introduction - final paragraph
**Methods**			
Study design	4	Present key elements of study design early in the paper	Page 4; Methods - Study design and setting
Setting	5	Describe the setting, locations, and relevant dates, including periods of recruitment, exposure, follow-up, and data collection	Page 4; Methods - Study design and setting
Participants	6(a)	Give the eligibility criteria, and the sources and methods of selection of participants. Describe methods of follow-up	Pages 4-5; Methods - Study population; Figure 2
Participants	6(b)	For matched studies, give matching criteria and number of exposed and unexposed	Not applicable: no matching was performed
Variables	7	Clearly define all outcomes, exposures, predictors, potential confounders, and effect modifiers. Give diagnostic criteria, if applicable	Pages 5-6; Methods - Data collection; Outcomes; ICG fluorescence angiography
Data sources/measurement	8*	For each variable of interest, give sources of data and details of methods of assessment (measurement). Describe comparability of assessment methods if there is more than one group	Page 5; Methods - Data collection; Outcomes; standardized institutional databases and definitions
Bias	9	Describe any efforts to address potential sources of bias	Pages 6 and 15-16; Methods - Statistical analysis; ICG fluorescence angiography; Discussion - limitations
Study size	10	Explain how the study size was arrived at	Pages 4 and 7; Methods - Study population; Results - Study cohort
Quantitative variables	11	Explain how quantitative variables were handled in the analyses. If applicable, describe which groupings were chosen and why	Page 6; Methods - Statistical analysis
Statistical methods	12(a)	Describe all statistical methods, including those used to control for confounding	Pages 6-7; Methods - Statistical analysis; Tables 3-4 and 7
Statistical methods	12(b)	Describe any methods used to examine subgroups and interactions	Pages 6-14; Methods - Statistical analysis; subgroup analyses by tumor location and ICG use
Statistical methods	12(c)	Explain how missing data were addressed	Page 6; Methods - Statistical analysis - missing data
Statistical methods	12(d)	If applicable, explain how loss to follow-up was addressed	Not applicable: fixed 30-day postoperative follow-up; no survival follow-up analyzed
Statistical methods	12(e)	Describe any sensitivity analyses	Page 7; Methods - Statistical analysis - bootstrap internal validation
**Results**			
Participants	13(a)	Report numbers of individuals at each stage of study—e.g., numbers potentially eligible, examined for eligibility, confirmed eligible, included in the study, completing follow-up, and analysed	Pages 5 and 7; Figure 2; Results - Study cohort
Participants	13(b)	Give reasons for non-participation at each stage	Pages 4-5 and 7; Methods - Study population; Results - Study cohort
Participants	13(c)	Consider use of a flow diagram	Page 5; Figure 2
Descriptive data	14(a)	Give characteristics of study participants (e.g., demographic, clinical, social) and information on exposures and potential confounders	Pages 7-8; Results - Study cohort; Tables 1-2
Descriptive data	14(b)	Indicate number of participants with missing data for each variable of interest	Page 6; Methods - Statistical analysis - missing data
Descriptive data	14(c)	Summarise follow-up time (e.g., average and total amount)	Not applicable: fixed 30-day postoperative follow-up
Outcome data	15	Report numbers of outcome events or summary measures over time	Pages 8-14; Results - Occurrence, clinical expression, management, consequences, and ICG use; Tables 3-8; Figures 3-6
Main results	16(a)	Give unadjusted estimates and, if applicable, confounder-adjusted estimates and their precision (e.g., 95% confidence interval). Make clear which confounders were adjusted for and why they were included	Pages 8-14; Results - main analyses; Tables 3-4 and 7; Figures 4 and 6
Main results	16(b)	Report category boundaries when continuous variables were categorized	Pages 7-13; Tables 1-7; predefined categories reported

Section/topic	Item No	Recommendation	Reported on page/section
			where used
Main results	16(c)	If relevant, consider translating estimates of relative risk into absolute risk for a meaningful time period	Pages 8 and 13; Tables 3 and 7 - absolute risk differences
Other analyses	17	Report other analyses done—e.g., analyses of subgroups and interactions, and sensitivity analyses	Pages 9 and 14; Model validation and ICG/subgroup descriptive analyses; Tables 4 and 8
**Discussion**			
Key results	18	Summarise key results with reference to study objectives	Page 14; Discussion - first paragraph
Limitations	19	Discuss limitations of the study, taking into account sources of potential bias or imprecision. Discuss both direction and magnitude of any potential bias	Pages 15-16; Discussion - limitations paragraph
Interpretation	20	Give a cautious overall interpretation of results considering objectives, limitations, multiplicity of analyses, results from similar studies, and other relevant evidence	Pages 14-16; Discussion - interpretation paragraphs; Conclusions
Generalisability	21	Discuss the generalisability (external validity) of the study results	Page 16; Discussion - clinical perspective and future studies; Conclusions
**Other information**			
Funding	22	Give the source of funding and the role of the funders for the present study and, if applicable, for the original study on which the present article is based	Page 16; Funding statement

*Give information separately for exposed and unexposed groups.

Note: An Explanation and Elaboration article discusses each checklist item and gives methodological background and published examples of transparent reporting. The STROBE checklist is best used in conjunction with this article. Information on the STROBE Initiative is available at http://www.strobe-statement.org
